# A cross-sectional study of depression with comorbid substance use dependency in pregnant adolescents from an informal settlement of Nairobi: drawing implications for treatment and prevention work

**DOI:** 10.1186/s12991-018-0222-2

**Published:** 2018-12-20

**Authors:** Eric Kimbui, Mary Kuria, Obadia Yator, Manasi Kumar

**Affiliations:** 1 Department of Psychiatry, School of Medicine, College of Health Sciences, University of Nairobi, P.O. Box 586-00100, Nairobi, Kenya; 2 Department of Psychiatry, School of Medicine, College of Health Sciences, University of Nairobi, P.O. Box 74846-00200, Nairobi, Kenya; 3 Department of Psychiatry, School of Medicine, College of Health Sciences, University of Nairobi, P.O. Box 799-00517, Nairobi, Kenya; 4 Department of Psychiatry, College of Health Sciences, University of Nairobi, P.O. Box 47074-00100, Nairobi, Kenya; 5 Department of Psychology, University College London, London, WC1E 7HB UK

**Keywords:** Pregnant adolescents, Alcohol abuse, Peer pressure, Partner and family support, Depression, Adversities

## Abstract

**Introduction:**

Adolescent pregnancy is a highly prevalent and significant public health problem in Kenya, and mental health needs of pregnant adolescent girls have been overlooked. Nearly, 50% of the world’s population comprises children and adolescents and 85% live in lower and middle-income countries.

**Objective:**

Pregnant adolescents were interviewed to ascertain certain social determinants of mental health such as social support, partner or parent support, and demographic profile and assessed for depression using EPDS and for severity of depression using BDI, and their alcohol abuse assessed using AUDIT.

**Methods:**

A cross-sectional descriptive study using a purposive sample of 212 pregnant adolescents visiting Kangemi Health Centre in Nairobi was conducted.

**Results:**

We found that 60.4% had depressive symptoms scores of 8 and above on EPDS, 51.9% were found to have severe depression score on BDI. About 26.9% were currently consuming alcohol. The more severely depressed participants were demonstrating greater alcohol use. Of the 110 pregnant adolescents who were severely depressed, 39 were currently consuming alcohol. We identified several alcohol use disorder factors associated with depression such as living with an alcoholic, ever and current use of alcohol, alcohol-related harm being experienced, being pressured to take alcohol. On our final multivariate logistic regression, we found that *being a student* (AOR 5.12, 95% CI 1.19–22.0, *P *= 0.028); *low family income* (between 5000 and 10,000 shillings) (AOR 0.22, 95% CI 0.09–0.56, *P *= 0.02); *unplanned pregnancy* (AOR 3.41, 95% CI 1.19–9.80, *P *= 0.023); *both negative and ambivalent attitudes of the unborn baby’s father, respectively* (AOR 8.72 95% CI 2.88–26.37 *P *< 0.001; AOR 4.26 95% CI 1.35–13.45, *P *= 0.013); *early age at sexual debut* (AOR 0.70, 95% CI 0.55–0.89, *P *= 0.003); and *ever used any psychoactive substances* (AOR 3.21, 95% CI 1.31–7.88, *P *= 0.011).

**Conclusion and recommendations:**

Alcohol abuse during pregnancy presents a significant public health burden and the associated health risks for the adolescent mother and her baby are enormous. We need to bolster screening for the comorbid disorders such as depression and substance use disorders, particularly alcohol in order to address mental health and psychosocial functioning of adolescents. The underlying adversities and sociocultural challenges need to be better understood and mechanisms that lead to comorbidities require further research. Depression interventions for Kenyan adolescents would need to embed screening, treatment and management of substance abuse.

## Introduction

**Fig. 1:**
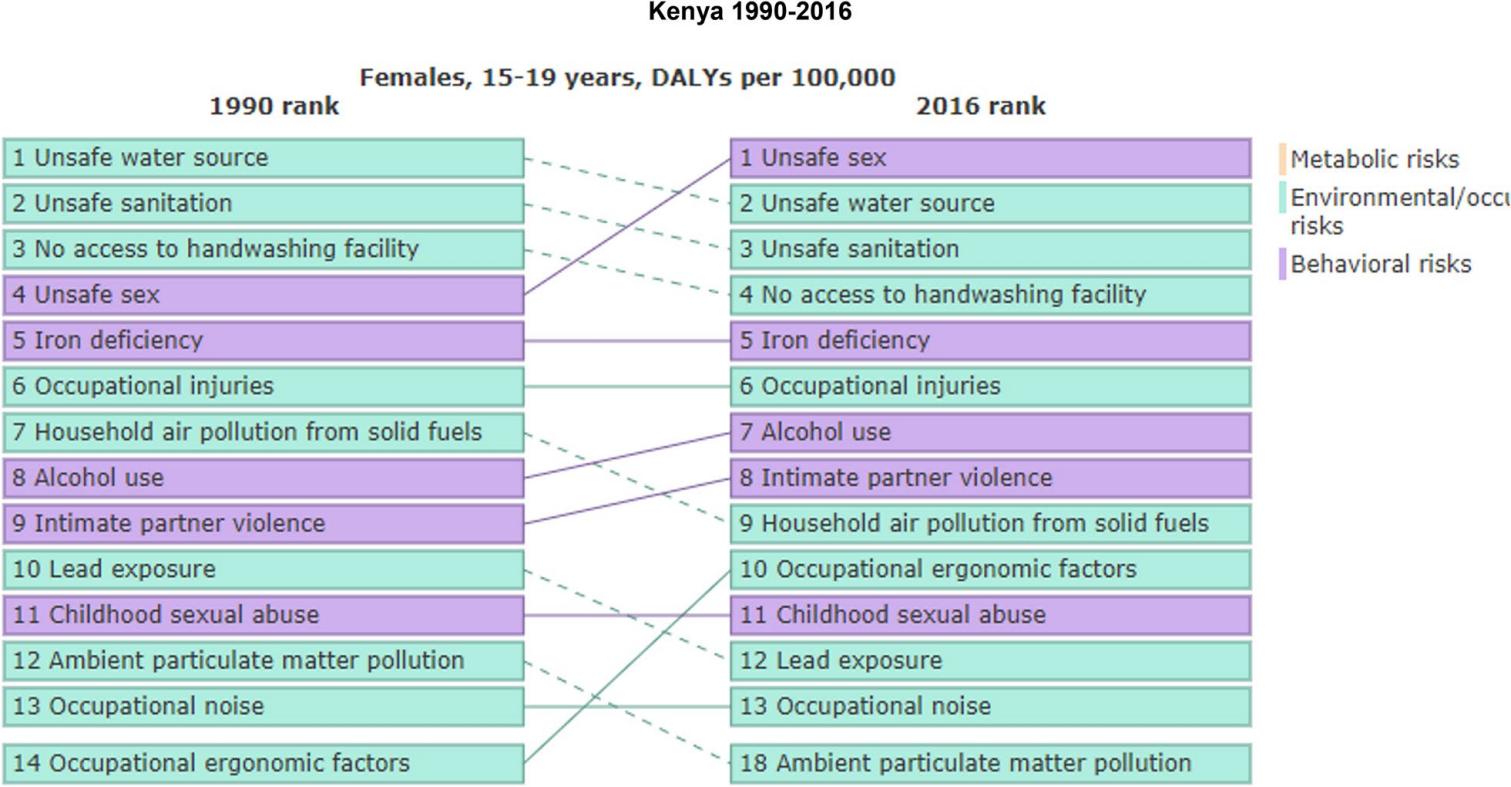
Comparison between DALYS-associated with females in Kenya from 1990 to 2016

Globally depression is the leading cause of disease burden in women of reproductive age. Prevalence is high during the perinatal period, with worldwide estimates of 11–18%. Perinatal maternal depression compromises health and well-being of both pregnant woman and infant. Health seeking behaviors and mental and substance use disorders in pregnant adolescents in East Africa have not been systematically documented. Unsafe sex, alcohol use disorders and intimate partner violence are amongst the key risk factors in adolescents of ages 15–19 years in Kenya [1] (see Fig. 1 indicating the burden of unsafe sex and alcohol use disorders based on Global Burden of Diseases 2016 estimate).

### Mental health burden in pregnant adolescents in Kenya and SSA

Health and educational status of women are key to physical, social and economic well-being of families and societies. In sub-Saharan Africa (SSA), 20–40% of teenagers are mothers or currently pregnant [2]. In Kenya, similar to other SSA countries, 22% of population were adolescents, 56% of adolescents had sex by age 16, only 45% of adolescents used condom during intercourse, 26% of girls give birth by age 18 (or 30% in urban regions), and only 42–51% of adolescents had comprehensive knowledge of HIV [3]. In Kenya, according to the 2008–2009 Kenya Demographic and Health Survey (KDHS), 18% of young women age 15–19 become pregnant: 15% are mothers and an additional 3% are pregnant with their first child. Another study conducted at Kenya Medical Research Institute (KEMRI) indicated that 30% of adolescent girls get pregnant in most urban centers; young motherhood is slightly more common in urban areas than in rural areas [4]. Pregnancies among girls less than 18 years of age have irreparable consequences and pose high development costs for communities, particularly in perpetuating the cycle of poverty. Two qualitative studies conducted in Uganda provide firsthand information about the various challenges that pregnant adolescents face. Lynne conducted a study where the respondents were pregnant adolescents, adolescent mothers, opinion leaders, in-charges of health unit, and traditional birth attendants and her findings revealed that pregnant adolescents faced domestic physical violence, and they were psychologically violated by parents and partners, and the community within which they lived [5]. Another longitudinal qualitative study in Uganda [6], Kaye explored what adolescent mothers perceive as their struggles during the period of transition from childhood to parenthood. Overall, young adolescents reported more anxiety, loss of self-esteem (when they conceived), difficulty in accessing financial, moral and material support from parents or partners and stigmatization by health workers when they sought care from health facilities [7, 8]. Specifically, depression, during and after pregnancy, is linked to multiple adverse health outcomes for mothers and children [9]. Adolescent pregnancy and motherhood present serious concerns for the mother, baby, community, and society at large [10] and the significant gaps in recognizing the health burden and psychosocial distress of this vulnerable group by community, health care and local policy stakeholders need mapping out [11].

### Depression and substance abuse pathogenesis

Depression along with other mood disorders, such as bipolar, is commonly found comorbidity with substance abuse disorders [12]. Depression indicates low mood; low energy levels and depleted functioning include reduced self-esteem and self-efficacy. Substance abuse or addiction disorders may emanate from depression if one is trying to boost mood or use peer pressure to relax from toxic and stressful environments. Substances like alcohol are depressants, so if a depressed person consumes such substances they may be aggravating their low mood, poor functioning and energy levels. There are various pathways used to understand the comorbidities associated with depression and substance abuse. In their pathogenesis, depression may lead to substance abuse and in some ways substance abuse may contribute towards depleted mood and self-efficacy. The self-medication hypothesis proposes that individuals treat their own depression by consuming alcohol and both feed into each other. Depression and substance use disorders in adolescents are both more likely in the presence of stressors and share many risk factors [13].

Environmental influences such as adverse childhood experiences play an important role in the development of psychiatric diseases to induce expression changes in important genes associated with MDD appearance of symptoms [14]. Low socioeconomic status and conflicts in the family have commonly been found to be a risk factor for later development of depression. Dropping out of school has also been found to be a significant predictor of later life substance abuse [15]. Comorbid depression and substance abuse are also known to have other social determinants in common such as poverty, racial/ethnic stigma, and inequitable treatment [15, 16]. The stress associated with these adversities triggers further distress [17]. Other studies have pointed out that the depressive episodes in adolescence are recurrent and may persist into adulthood if the contributing factors remain unchanged [18]. The presence of either disorder doubled the risks of the second disorder and there are causal mechanisms linking the two. The potential mechanisms underlying these causal linkages include neurophysiological and metabolic changes resulting from exposure to alcohol [19]. There are also suggestions that these comorbidities are significantly more associated with moderate to severe depressive symptoms than with mild depressive symptoms [20]. Psychosocial impairment tends to be more severe in depressed adolescents with comorbid substance use disorders than in those without [21].

#### Perinatal depression and associated risks in adolescence

A number of studies have pointed to associated risks such as fetal alcohol syndrome and sudden infant death syndrome due to comorbid substance abuse and mood disorders [22]. Pre-term births, low birth weight and other adverse obstetric outcomes are also associated with both substance abuse and depression [23]. One study also pointed to the challenges faced by antepartum women with mental illness in being able to quit substances and pointed to the challenges in prioritizing mental illness over addiction as health inequity increased amongst this vulnerable group [24]. Connelly et al. have argued that depression screening alone may not be the correct response to perinatal disorders and that we need to integrate adverse experiences such as IPV and substance abuse. Children, adolescents and young people living in poverty in Kenya are more likely than others to be depressed [25]. In two exploratory qualitative studies on pregnant adolescents, we found that similar issues were raised [8, 26]. By 2010, adolescents in sub-Saharan Africa accounted for 16% of the world total. The greatest increase in pregnancy among adolescent girls less than 18 years of age over the next 20 years is likely to happen in SSA [27], and Kenya is one of the top five SSA countries where the uptake of contraceptives is still low and the prevalence of adolescent pregnancy continues to remain high. UNFPA projects that the potential number for SSA could eventually equal or surpass the one for South Asia around 2025–2030, with a total of 3 million adolescents who could have had their first birth before age 15 in this region [28]. Additionally, adolescents lack comprehensive knowledge about the risks stemming from early sexual activity, as well as appropriate measures to prevent infection and pregnancy.

The aim of this study was to provide preliminary links between depression and substance use, namely alcohol abuse, and explore psychosocial correlates such as partner and family support, sexual debut and critical pointers such as whether the early pregnancy was planned, wanted or not.

## Method

### Study design, sample and setting

Due to the limited populations of pregnant adolescents visiting attending Kangemi Health Centre which is a community-based facility in Nairobi, we conducted a cross-sectional descriptive study using purposive sampling to assess prevalence of depression, substance abuse and associated risk factors. The facility attends to patients on a daily basis throughout the week and on average the adolescent population visits range from 10 to 16 visits per day. Kangemi is an area made up mostly of informal settlements located on the outskirts of Nairobi, on Waiyaki Way: a highway that connects Nairobi city and Naivasha town. It is about 1.6 km^2^ in size with an estimated population of more than 100,000 residents. It borders Loresho and Kibagare to its north, West lands to its west, Mountain View to its east and Kawangware: another large informal settlement to its south.

Through convenient sampling, we recruited 212 participants aged 14–18 years using prevalence rate of 26% [3]. The sample size was estimated using Cochran formula where alpha was kept at *P *< 0.05 [29].

### Study procedures

The study was approved by Kenyatta National Hospital and University of Nairobi Ethics Review Committee (approval no. P530/07/2016) and also by the Nairobi county government Ref: HRD/22/2612/HO/2016. We started our study by explaining to the participants the purpose of the study and most importantly that participation was on voluntary basis. They were also assured of anonymity and confidentiality considering that adolescent pregnancy was noted to be associated with promiscuity in this low resource setting. Those who agreed to sign a written informed consent were assigned code numbers to conceal their identity. Once data collection was completed, the assessment instruments were kept in a lockable cabinet to enhance confidentiality. Data collection was done daily on Monday to Saturday from the beginning of October 2016 until mid-December 2016. All participants who were introduced to the study accepted to participate.

Assessment was done in one of the consultation rooms within the ANC clinic where all the participants were administered a researcher-designed demographic and psychosocial risk factors questionnaire, the Alcohol Use Disorders Identification Test (AUDIT), the Edinburgh Postnatal Depression Screen (EPDS) and the Becks Depression Inventory II (BDI II). Those with an AUDIT cutoff score of 8+ were referred to the Center for Substance Abuse Treatment (CSAT) at Mathare National Teaching and those with BDI cutoff point of 22+ were referred to the Kenyatta National Hospital Youth/Reproductive Health Centre. The researcher also offered psychosocial support for those in distress due to other associated risk factors.

## Measures

### Research designed questionnaire

We inquired on specific socio-demographic characteristics such as age, parity, level of education, income and presence of social support either from partner, mother, father, friend or religious institutions. It also had a few questions on previous psychoactive substance use and past illnesses. We included a few questions on antenatal care and obstetric history.

### Perinatal depression screening tool

We used a Kiswahili translated version of the Edinburgh Postnatal Depression Screen. EPDS is appropriate instrument for assessing prenatal depression in Kenya and sub-Saharan Africa (SSA) [30, 31]. The psychometric properties of the EPDS in primary health care were: 86% sensitivity (correctly identifying true cases), 78% specificity (correctly identifying people without the condition) and 73% positive predictive value (proportion of respondents scoring positive in the test who had a mental disorder diagnosed by clinical interview) [30]. A Kiswahili translated version of EPDS was well received by this group [32]. A cutoff of 8+ or 10 depicts probable minor depressive symptoms and a cutoff of 13+ signifies probable major depression. For this study, we used a cutoff of 8+ to be able to know the extent of perinatal depression among pregnant adolescents.

### Depression diagnosis and severity assessment

In our study, any participant scoring 8 or above on EPDS was subjected Becks Depression Inventory II to diagnose and classify depression severity. The BDI (II) is a 21-item self-report rating inventory that measures characteristic attitudes and symptoms of depression [33, 34]. The BDI takes approximately 10 min to complete. The BDI demonstrates high internal consistency, with alpha coefficients of 0.86 and 0.81 for psychiatric and non-psychiatric populations, respectively [35]. Our study further supports the BDI (II) as a viable and reliable measure for identifying probable cases of Depressive disorders among adolescents. We scored the BDI-II by summing the ratings for the 21 items. Each item is rated on a 4-point scale ranging from 0 to 3. The maximum total score is 63. We paid special attention the scoring of the Changes in Sleeping Pattern (Item 16) and Changes in Appetite (Item 18) items. Each of these items contains seven options rated, in order, 0, 1a, 1b, 2a, 2b, 3a, 3b, to differentiate between increases and decreases in behavior or motivation. If a higher rated option is chosen by the respondent, the presence of an increase or decrease in either symptom should be clinically noted for diagnostic purposes. On each item, participants were asked to choose the statement that best describes their attitude towards the item.

### Alcohol use screening tool

The Alcohol Use Disorders Identification Test is a 10-item was developed by World Health Organization (WHO) to assess alcohol consumption, drinking behaviors, and alcohol-related problems [36, 37]. It has both a clinician-administered version and a self-report. In our study, we used the self-report version which is easy and takes less than 5 min to complete. The total AUDIT score is an indicator of the level of alcohol use with scores: 0–7 for low risk, 8–15 for risky or hazardous levels, 16–19 for high risk or harmful levels, 20 or more for high-risky or dependence. Our study used a cutoff of 8+ to identify those with alcohol use disorder.

## Data analysis

We used SPSSv21 to analyze the data once the data was coded, entered and de-identified [38]. Descriptive statistics were employed to estimate the prevalence of dependent variables such as current alcohol consumption, dependence, antenatal depression and depression severity as well as the participant’s socio-demographic characteristics being independent variables. Mean prevalence rates and their respective 95% CI were estimated including subgroups. Univariate associations of current alcohol use and depression and each of the following variables were estimated using bivariate logistic regression which were first fitted to identify potential confounding factors and variables with a *P* value less than 0.2 which were entered to multiple hierarchical logistic regression models using enter method to identify factors associated factors with current alcohol use and depression in 4 blocks. Adjusted odds ratio with its 95% confidence interval was calculated to report the strength and significance of the association. All tests were two sided and statistical significance was declared at *P *< 0.05.

## Results

### Socio-demographic characteristics of the respondents

Out of the 212 participants, 50% (*N *= 106) of the pregnant adolescents were 18 years old and remaining were between ages 16–17 years. The mean age of our study participants was 17.3 years (SD = 1.9) and almost three quarters (72.2%) of the participants were unmarried and 29.7% married or cohabiting with a partner. About 48.6% (*N *= 103) of the participants had had primary education and 51.4% (*N *= 109) had attained some level of secondary education. About 68.9% were out of school and not employed and, therefore, financially dependent on parents, 15.6% (*N *= 33) were currently studying and another 15.6% were gainfully employed.

More than three quarters (77.8%) of the participants lived in a household where the monthly income was less than 10,000 Kenyan shillings (≈ 100 USD) per month with a third (31.6%) of them living in a household where the family income was less than 5000 Kenyan shillings (≈ 50 USD). The majority (42%) of the adolescent participants currently lived with persons other than their parents, partners, or friends making us wonder if they may have been employed as domestic workers and living with their employers (see Table 1).

**Table 1:** Socio-demographic characteristics of the respondents

Variable	Category	Frequency (*N *= 212)	Percent (%)
Age in years	16 years and below	34	16.0
	17 years	72	34.0
	18 years	106	50.0
Age in years	(Mean, SD, range)	(17.3, 1.92, 14–18)	
Marital status	Single	153	72.2
	Married/with partner	59	27.8
Level of education	Primary school	103	48.6
	Secondary/high school	109	51.4
Current occupation	Student	33	15.6
	Employed	33	15.6
	Unemployed	146	68.9
Religion	Christian	207	97.6
	Other	5	2.4
Family monthly income (Ksh.)	< 4999	67	31.6
	5000–9999	98	46.2
	10,000–34,999	44	20.8
	> 35,000	3	1.4
Persons living with	Parents	66	31.1
	Spouse	57	26.9
	Friends/alone	7	3.3
	Others	82	38.7
Psychological history and psychiatric characteristics of the respondents			
Attitude of the father towards the pregnancy	Positive	87	41.0
	Negative	78	36.8
	Ambivalent	44	20.8
	Not told about pregnancy	3	1.4
Presence of social support (partner; mother; friend; church etc.)	No	47	22.2
	Yes	165	77.8
Family member ever been treated for a mental illness, e.g., depression or committed suicide?	No	181	85.4
	Yes	31	14.6
Age at sexual debut (years)	< 11	13	6.1
	11–14	44	20.8
	15–18	155	73.1
Age at sexual debut	(Mean, SD, range)	(15.3, 1.9, 9–18)	
Experienced intimate partner violence in pregnancy	No	198	93.4
	Yes	14	6.6
EPDS categories	Normal	84	39.6
	Abnormal	128	60.4
EPDS scores	(Mean, SD, range)	(12.3, 7.7, 0–27)	
BECK depression categories	Normal	23	10.8
	Mild	23	10.8
	Moderate	56	26.4
	Severe	110	51.9
BECK depression	(Mean, SD, range)	(29.4, 12.2, 4–60)	

### Psychological history and psychiatric characteristics of respondents

The mean EPDS score was 12.3 (SD = 7.7) with most (60.4%) of the participants exhibiting a score of 8 and above whereas in terms of severity of depression assessment using BDI II, the mean score was found to be 29.4 (SD = 12.2). Out of the 212 participants, 10.8% (*N *= 23) had no depression, 10.8% (*N *= 23) were found to have mild depression, 26.4% (*N *= 56) were found to have moderate depression and 51.9% (*N *= 110) were found to have severe depression.

Our study found out that 41% of the partners of the pregnant adolescents had a positive attitude towards the pregnancy, 36.8% had negative attitude and about 1.4% had not disclosed the pregnancy to their partners. A majority (77.8%) of the adolescents received some social support from either their partner, mother, friend and even from their community and 22.2% reported an absence of support from these relationships and sources. Almost three quarters (73.1%) of the adolescents in this study had an early sexual debut while between the ages of 15 to 18 years with the mean age of initiation of first sexual activity being 15.3 years (SD = 1.9). Intimate partner violence was reported to be low (6.6%) (see Table 2).

**Table 2:** Obstetric history of the respondents

Variable	Category	Frequency (*N *= 212)	Percent (%)
History of chronic illness	No	197	92.9
	Yes	15	7.1
Currently on medication	No	168	79.2
	Yes	44	20.8
Week of gestation of first ANC visit (weeks)	< 12	34	16.0
	12–28	176	83.0
	> 28	2	0.9
Pregnancy unplanned	No	33	15.6
	Yes	179	84.4
Parity	First pregnancy	208	98.1
	One previous pregnancy	4	1.9
Is pregnancy wanted?	No	40	18.9
	Yes	172	81.1

### Obstetric history of the respondents

Most (83.0%) of these pregnant adolescents had attended their initial ANC visit between 12 and 28 weeks of gestation. The majority (84.4%) had not planned for the pregnancy but despite this a large majority (81.1%) wanted to bring the pregnancy to fruition. Despite their young age range of 11–18 years, four of the participants had had a previous early pregnancy (see Table 3).

**Table 3:** Alcohol drug and substance use of the respondents

Variable	Category	Frequency (*N *= 212)^a^	Percent (%)
Living with anyone who is a problem drinker or alcoholic or who uses street drugs	No	173	81.6
	Yes	39	18.4
Ever consumed alcohol at any time in your life?	No	119	56.1
	Yes	93	43.9
Age at first consumption (*N *= 92), years	< 11	7	7.6
	11–14	24	26.1
	15–18	61	66.3
Currently consuming alcohol	No	155	73.1
	Yes	57	26.9
Consumption at a hazardous level (*N *= 57)	No	41	71.9
	Yes	16	28.1
Alcohol dependence (*N *= 57)	No	18	31.6
	Yes	39	68.4
Audit scores	(Mean, SD, range)	(2.5, 5.6, 0–34)	
Alcohol-related harm is already being experienced	No	155	73.1
	Yes	57	26.9
Ever been pressured into using alcohol or other substances by your friends or peers?	No	140	66.0
	Yes	72	34.0
Currently or have you ever smoked Cigarettes?	No	207	97.6
	Yes	5	2.4
Ever used any other psychoactive substance	No	194	91.5
	Yes	18	8.5

*SD* standard deviation

^a^Total number of respondents is 212 unless otherwise indicated on the variable

### Alcohol use by the respondents

With regard to alcohol consumption, the mean AUDIT score was 2.5 (SD = 5.6). Ninety-three participants out of the 212 (43.9%) had consumed alcohol once and majority (66.3%) of the adolescents had done so between ages of 15–18 years. Of those who had ever consumed alcohol, seven participants (7.6%, *N *= 93) shared that they had consumed alcohol when they were less than 11 years old. Twenty-seven percent of the participants (*N *= 57) were currently consuming alcohol despite being pregnant with majority (68.4%) of this specific group showing features of alcohol dependence. About 8.5% reported using other psychoactive substances. Additionally, about 18.4% indicated that they lived with someone who was an alcoholic and used street drugs (see Tables 4 and 5). About 32.8% of those who were consuming alcohol were found to have clinically elevated scores on EPDS. From those who were found with alcohol use disorder, 21 participants (53.8%) were living with someone who was a problem drinker (*χ*^2^(1,212) = 17.67; *P *< 0.000) and 15 (83.3%) of the participants were using other psychoactive substances (χ^2^(1,212) = 31.88; *P *= 0.000). Table 5 presents disaggregated alcohol use patterns disaggregated by the presence of depression.

**Table 4:** Drug and substance use factors associated with depression among the respondents

Variable	Category	Overall	Depression		OR (95% CI)	Group differences
			No	Yes		
Live with anyone who is a problem drinker or alcoholic or who uses street drugs	No	173 (81.6)	43 (24.9)	130 (75.1)	*Reference*	(*χ*^2^(1,212) = 5.52; *P = 0.019*)
	Yes	39 (18.4)	3 (7.7)	36 (92.3)	3.97 (1.16–13.54)	
Ever consumed alcohol at any time in your life?	No	119 (56.1)	32 (26.9)	87 (73.1)	*Reference*	(*χ*^2^(1,212) = 4.31; *P = 0.038*)
	Yes	93 (43.9)	14 (15.1)	79 (84.9)	2.08 (1.03–4.17)	
Age at first consumption, years	< 11	7 (7.6)	0 (0.0)	7 (100.0)	*Reference*	(*χ*^2^(2,92) = 1.30; *P *= 0.522)
	11–14	24 (26.1)	4 (16.7)	20 (83.3)	UD	
	15–18	61 (66.3)	9 (14.8)	52 (85.2)	UD	
Currently consuming alcohol	No	155 (73.1)	38 (24.5)	117 (75.5)	*Reference*	(*χ*^2^(1,212) = 2.70; *P = 0.101*)
	Yes	57 (26.9)	8 (14.0)	49 (86.0)	1.99 (0.87–4.57)	
Audit scores	Normal	184 (86.8)	43 (23.4)	141 (76.6)	*Reference*	(*χ*^2^(1,212) = 2.29; *P = 0.130*)
	Abnormal	28 (13.2)	3 (10.7)	25 (89.3)	2.54 (0.73–8.83)	
Consumption at a hazardous level	No	41 (71.9)	6 (14.6)	35 (85.4)	*Reference*	(*χ*^2^(1,57) = 0.04; *P *= 0.835)
	Yes	16 (28.1)	2 (12.5)	14 (87.5)	1.20 (0.22–6.68)	
Alcohol dependence	No	18 (31.6)	6 (33.3)	12 (66.7)	*Reference*	(*χ*^2^(1,57) = 8.12; *P = 0.004*)
	Yes	39 (68.4)	2 (5.1)	37 (94.9)	9.25 (1.64–52.06)	
Alcohol-related harm is already being experienced	No	155 (73.1)	39 (25.2)	116 (74.8)	*Reference*	(*χ*^2^(1,212) = 4.07; *P = 0.044*)
	Yes	57 (26.9)	7 (12.3)	50 (87.7)	2.40 (1.01–5.73)	
Ever been pressured into using alcohol or other substances by your friends or peers	No	140 (66.0)	36 (25.7)	104 (74.3)	*Reference*	(*χ*^2^(1,212) = 3.91; *P = 0.048*)
	Yes	72 (34.0)	10 (13.9)	62 (86.1)	2.15 (1.00–4.63)	
Currently or have ever smoked cigarettes	No	207 (97.6)	46 (22.2)	161 (77.8)	*Reference*	(*χ*^2^(1,212) = 1.42; *P *= 0.234)
	Yes	5 (2.4)	0 (0.0)	5 (100.0)	UD	
Have you ever used any other psychoactive substance/s	No	194 (91.5)	42 (21.6)	152 (78.4)	*Reference*	(*χ*^2^(1,212) = 0.00; *P *= 0.955)
	Yes	18 (8.5)	4 (22.2)	14 (77.8)	0.97 (0.30–3.09)	

The values in italic represents significant differences (*P* < 0.05) between depression levels and alcohol use

*OR* odds ratio, *CI* confidence interval, *Ref.* reference category, *UD* undetermined

**Table 5:** Prevalence of alcohol consumption disaggregated by the presence of depression

	Overall (*N *= 212)		Not depressed (*N *= 84)		Depressed (*N *= 128)	
	*n* (%)	(95% CI)	*n* (%)	(95% CI)	*n* (%)	(95% CI)
Currently consuming alcohol (*N *= 212)						
Yes	57 (26.9)	(20.8–32.5)	15 (17.9)	(10.7–27.4)	42 (32.8)	(25.0–40.6)
Ever consumed alcohol (*N *= 212)						
Yes	93 (43.9)	(37.3–50.5)	30 (35.7)	(25.0–46.4)	63 (49.2)	(40.6–57.8)
Age at which it was first taken (*N *= 91), years						
< 11	6 (6.6)	(2.2–12.1)	1 (3.6)	(0.0–10.7)	5 (7.9)	(1.6–14.3)
11–14	24 (26.4)	(17.6–36.3)	6 (21.4)	(7.1–35.7)	18 (28.6)	(17.5–39.7)
15–18	61 (67.0)	(57.1–75.8)	21 (75.0)	(57.1–89.3)	40 (63.5)	(50.8–74.6)
Type of alcohol (*N *= 91)						
Beer, wine or bottled spirits	59 (64.8)	(54.9–74.7)	19 (67.9)	(50.0–85.7)	40 (63.5)	(50.8–74.6)
Local brews	29 (31.9)	(22.0–41.8)	8 (28.6)	(10.7–46.4)	21 (33.3)	(22.2–44.4)
Both beer, wine and local brews	3 (3.3)	(0.0–7.7)	1 (3.6)	(0.0–10.7)	2 (3.2)	(0.0–7.9)
How often do you have a drink containing alcohol? (*N *= 212)						
Never	155 (73.1)	(73.1–67.5)	69 (82.1)	(82.1–73.8)	86 (67.2)	(67.2–57.8)
Monthly or less	35 (16.5)	(16.5–11.3)	9 (10.7)	(10.7–4.8)	26 (20.3)	(20.3–13.3)
2–4 times a month	12 (5.7)	(5.7–2.8)	5 (6.0)	(6.0–1.2)	7 (5.5)	(5.5–2.3)
2–3 times a week	8 (3.8)	(3.8–1.4)	1 (1.2)	(1.2–0.0)	7 (5.5)	(5.5–2.3)
4 or more times a week	2 (0.9)	(0.9–0.0)	0 (0.0)	(0.0–0.0)	2 (1.6)	(1.6–0.0)
Audit scores (*N *= 57)						
0–7 normal	29 (50.9)	(38.6–63.2)	10 (66.7)	(40.0–93.3)	19 (45.2)	(31.0–59.5)
8 and above-harmful and hazardous use	28 (49.1)	(36.8–61.4)	5 (33.3)	(6.7–60.0)	23 (54.8)	(40.5–69.0)
Alcohol dependence (*N *= 57)						
Yes	39 (68.4)	(56.2–78.9)	6 (40.0)	(20.0–66.7)	33 (78.6)	(66.7–90.5)
Alcohol-related harm already being experienced (*N *= 57)						
Yes	51 (89.5)	(80.7–96.5)	13 (86.7)	(66.7–100.0)	38 (90.5)	(81.0–97.6)
Hazardous alcohol use (*N *= 57)						
Yes	16 (28.1)	(15.8–40.4)	3 (20.0)	(0.0–40.0)	13 (31.0)	(16.7–45.2)

### Multivariate analyses and logistic regression model

On multivariate analyses, the following alcohol and substance uses related behaviors were found to be positively associated with depression, *living with anyone who was an alcoholic/used drugs* was found to be significant (χ^2^ = 5.52*, P *= 0.019, OR 1.16–13.45), *ever consumed alcohol* (χ^2^ = 4.31, *P *= 0.038, OR 1.03–4.17), *alcohol dependence* (χ^2^ = 8.12, *P *= 004), *alcohol-related harm already being experienced* (χ^2^ = 4.07, *P *= 0.044), and ever being pressured into consuming alcohol or substances by peers or friends (χ^2^ = 3.91, *P *= 0.048*)* (see Table 6).

**Table 6:** Levels of depression and alcohol use

	Levels of depression				Group differences
	Normal (*N *= 23)	Mild (*N *= 23)	Moderate (*N *= 56)	Severe (*N *= 110)	
Currently consuming alcohol					
No	18 (11.6)	20 (12.9)	46 (29.7)	71 (45.8)	(*χ*^2^(3,212) = 9.0; *P = 0.030*)
Yes	5 (8.8)	3 (5.3)	10 (17.5)	39 (68.4)	
Frequency of consumption					
Never	18 (11.6)	20 (12.9)	46 (29.7)	71 (45.8)	(*χ*^2^(12,212) = 14.7; *P *= 0.256)
Monthly or less	2 (5.7)	2 (5.7)	7 (20.0)	24 (68.6)	
2 to 4 times a month	3 (25.0)	0 (0.0)	2 (16.7)	7 (58.3)	
2 to 3 times per week	0 (0.0)	1 (12.5)	1 (12.5)	6 (75.0)	
> 4 times a week	0 (0.0)	0 (0.0)	0 (0.0)	2 (100.0)	
Audit scores					
Normal	22 (12.0)	21 (11.4)	52 (28.3)	89 (48.4)	(*χ*^2^(3,212) = 7.1; *P *= 0.069)
Abnormal	1 (3.6)	2 (7.1)	4 (14.3)	21 (75.0)	
Alcohol-related harm is already being experienced					
No	19 (12.3)	20 (12.9)	45 (29.0)	71 (45.8)	(*χ*^2^(3,212) = 8.9; *P = 0.031)*
Yes	4 (7.0)	3 (5.3)	11 (19.3)	39 (68.4)	
Alcohol dependence					
No	3 (16.7)	3 (16.7)	3 (16.7)	9 (50.0)	(*χ*^2^(3,57) = 9.7; *P *= *0.021*)
Yes	2 (5.1)	0 (0.0)	7 (17.9)	30 (76.9)	
Hazardous alcohol consumption					
No	5 (12.2)	1 (2.4)	8 (19.5)	27 (65.9)	(*χ*^2^(3,57) = 4.6; *P *= 0.201)
Yes	2 (0.0)	2 (12.5)	2 (12.5)	12 (75.0)	
Ever consumed alcohol at any time in your life					
No	15 (12.6)	17 (14.3)	35 (29.4)	52 (43.7)	(*χ*^2^(3,212) = 8.2; *P = 0.043)*
Yes	8 (8.6)	6 (6.5)	21 (22.6)	58 (62.4)	
Age at which it was consumed, years					
< 11	0 (0.0)	0 (0.0)	3 (42.9)	4 (57.1)	(*χ*^2^(6,92) = 3.2; *P *= 0.782)
11–14	2 (8.3)	2 (8.3)	4 (16.7)	16 (66.7)	
15–18	6 (9.8)	3 (4.9)	14 (23.0)	38 (62.3)	
Type of alcohol have you consumed in the past					
Beer, wine or bottled spirits	4 (6.8)	4 (6.8)	10 (16.9)	41 (69.5)	(*χ*^2^(6,91) = 4.8; *P *= 0.566)
Local brews	4 (13.8)	1 (3.4)	9 (31.0)	15 (51.7)	
Both beer, wine and local brews	0 (0.0)	0 (0.0)	1 (33.3)	2 (66.7)	
Ever been pressured into using alcohol or other substances by your friends or peers					
No	17 (12.1)	19 (13.6)	47 (33.6)	57 (40.7)	(*χ*^2^(3,212) = 21.4; *P < 0.001)*
Yes	6 (8.3)	4 (5.6)	9 (12.5)	53 (73.6)	

Note: Chi-square test on Depression levels (BDI) by alcohol use factors (AUDIT); The values in italic represents significant differences (*P* < 0.05) between depression levels and alcohol use

Group comparisons in terms of depression severity and alcohol-related factors revealed a positive association χ^2^(3,212) = 9.0; *P *= 0.030) *on whether or not alcohol was consumed* and amongst those who were consuming alcohol those with severe depression reported highest consumption, followed by moderately depressed and then the remaining two categories. Significant differences were also found on depression severity and *whether alcohol-associated significant harm* was being experienced. Amongst those who reported experiencing alcohol-related harm (χ^2^(3,212) = 8.9; *P *= 0.031), those who were moderately and severely depressed had the highest proportion of alcohol-related harm. Significant associations were also found on *whether alcohol was ever consumed during lifetime* χ^2^(3,212) = 8.2; *P *= 0.043) and *whether peers or friends pressured to take alcohol* χ^2^(3,212) = 21.4; *P *< 0.001); in both these factors, there were significantly higher proportion of participants with severe depression who had both consumed alcohol and had been pressured by peers and friends (see Table 7).

**Table 7:** Multivariate logistic regression on factors associated with depression use among the participants (*P *< 0.2)

Variable	Category	OR (95% CI)	AOR	95% CI	*P*-value
Marital status	Single	3.61 (1.82–7.17)	0.16	(0.01–3.87)	0.260
	Married/with partner	*Ref.*		*Ref.*	
Highest level of education	Primary school	0.53 (0.27–1.03)	0.68	(0.29–1.57)	0.368
	Secondary	*Ref.*		*Ref.*	
Occupation	Student	2.70 (0.77–9.42)	5.12	(1.19–22.00)	*0.028*
	Employed	0.47 (0.21–1.06)	0.45	(0.16–1.30)	0.142
	Unemployed	*Ref.*		*Ref.*	
Family monthly income	< 4999	*Ref.*		*Ref.*	
	5000–9999	0.41 (0.18–0.94)	0.22	(0.09–0.56)	*0.002*
	10,000–34,999	0.60 (0.22–1.66)	0.60	(0.20–1.79)	0.361
	> 35,000	0.31 (0.03–3.78)	2.00	(0.14–29.25)	0.614
Persons living with	Parents	*Ref.*		*Ref.*	
	Spouse	0.35 (0.16–0.80)	0.41	(0.02–8.44)	0.562
	Friends/alone	0.56 (0.10–3.21)	0.73	(0.09–6.04)	0.766
	Others	1.60 (0.64–3.98)	1.00	(0.38–2.62)	0.994
History of chronic illness	No	*Ref.*		*Ref.*	
	Yes	*α* (0.00–)	4.35	(0.66–28.48)	0.125
Currently on any medication	No	*Ref.*		*Ref.*	
	Yes	0.44 (0.21–0.91)	1.54	(0.58–4.05)	0.384
Pregnancy unplanned	No	*Ref.*		*Ref.*	
	Yes	2.88 (1.30–6.36)	3.41	(1.19–9.80)	*0.023*
Pregnancy wanted	No	6.53 (1.51–28.20)	3.24	(0.83–12.74)	0.092
	Yes	*Ref.*		*Ref.*	
Attitude of the father towards the pregnancy	Positive	*Ref.*		*Ref.*	
	Negative	9.83 (3.61–26.77)	8.72	(2.88–26.37)	*< 0.001*
	Ambivalent	5.25 (1.88–14.63)	4.26	(1.35–13.45)	*0.013*
	Not told	1.35 (0.12–15.42)	0.29	(0.01–6.67)	0.436
Presence of social support	No	5.17 (1.53–17.51)	1.48	(0.48–4.58)	0.496
	Yes	*Ref.*		*Ref.*	
Age at sex debut	Mean (SD)	0.73 (0.58–0.92)	0.70	(0.55–0.89)	*0.003*
Live with anyone who is a problem drinker or alcoholic or who uses street drugs	No	*Ref.*		*Ref.*	
	Yes	3.97 (1.16–13.54)	1.23	(0.42–3.55)	0.706
Ever consumed alcohol at any time in life	No	*Ref.*		*Ref.*	
	Yes	2.08 (1.03–4.17)	1.15	(0.41–3.26)	0.787
Alcohol-related harm being experienced	No	*Ref.*		*Ref.*	
	Yes	2.40 (1.01–5.73)	1.08	(0.32–3.70)	0.901
Ever used any other psychoactive substance	No	*Ref.*		*Ref.*	
	Yes	0.97 (0.30–3.09)	3.21	(1.31–7.88)	*0.011*

The values in italic represents significant differences (*P* < 0.05) between depression levels and alcohol use

*OR* odds ratio, *AOR* adjusted odds ratio, *CI* confidence interval, *Ref.* reference category; *α* > 10,000

On the multivariate logistic regression, the following factors were significantly associated with elevated depressive symptoms: *being a student* (AOR 5.12, 95% CI 1.19–22.0, *P *= 0.028); *low family income* (between 5000 and 10,000 shillings) (AOR 0.22, 95% CI 0.09–0.56, *P *= 0.02); *unplanned pregnancy* (AOR 3.41, 95% CI 1.19–9.80, *P *= 0.023); *both negative and ambivalent attitudes of the unborn baby’s father, respectively* (AOR 8.72 95% CI 2.88–26.37 *P *< 0.001; AOR 4.26 95% CI 1.35–13.45, *P *= 0.013); *early age at sexual debut* (AOR 0.70, 95% CI 0.55–0.89, *P *= 0.003); and *ever used any psychoactive substances* (AOR 3.21, 95% CI 1.31–7.88, *P *= 0.011) (see Table 7).

These dense findings convey that the risk factors for both depression and alcohol and substance abuse might be common.

## Discussion

The findings of our work are in tandem with studies that have suggested a link between depression and substance abuse in adolescents [39]. The associated risk factors such as low-income status, pregnancy while at school, use of psychoactive substances, early sexual debut and poor support from adolescent male partner earmarked in the literature are precursors to the development of mental health problems in peripartum adolescents [40, 41]. Slightly, more than a quarter of the adolescents in our study were already experiencing alcohol-related harm and this may have already impacted the unborn baby. Bulletin of the World Health Organization has advocated greater awareness about the medical, social and emotional burden associated with fetal alcohol syndrome in SSA [42], another challenging issue surrounding unplanned adolescent pregnancies about which little is known.

The trio of depressive disorder, substance use disorder and adolescent pregnancy in a low-income context are a lethal combination; they are likely to result in numerous complications for the unborn baby and the mother [43]. In our sample, it was difficult to tell whether the depression was secondary to alcohol use disorder or vice versa or attributed to unplanned, unexpected pregnancy. Prolonged substance abuse especially alcohol use is known to reduce brain serotonin levels resulting in depression or increasing its severity. Further alcohol use during pregnancy is known to cause fetal alcohol syndrome [44]. In our sample, about 7 participants were consuming alcohol by the time they were 11 years old and another 24 when they were between 11 and 14 years old. Out of these two groups, all 7 and about 20 were found to be depressed (see Table 4).

### Depression pathogenesis-poor support, partner rejection and adversities lead to substances

Almost 32% of our participants belonged to households who were living under 5000 shillings a month (about 50 dollars a month) and the food insecurity and economic adversities must be considerable. Nearly half of the sample comprised adolescents under 18 years of age with 16% who were pregnant before they were 16 years old. Almost half of our participants had dropped out of school after completing primary education and that clearly is an impediment in future employability and in securing a future for themselves and their unborn baby. In the Kenyan cultural context, all social protection could be jeopardized if a pregnant adolescent is struggling with mental health problems or is not fully functional. We have discussed these cultural and structural barriers at length in another paper [8, 26]. Pregnant adolescents are likely to suffer rejection and stigma, with little social support from family and peers in a setup where abortion is illegal. Our finding that the adolescent male partner’s negative and ambivalent attitude could aggravate depression and trigger substance use as a result of social exclusion and ostracization is a noteworthy finding. The absence of male partner support or if the pregnancy was borne out of an abuse or non-serious non-committal relationship context could cause more distress and anxieties. The emotional disarray in the absence of partner or familial support is tremendous and a large number of those who were consuming alcohol were doing so in the company of peers. Few non-governmental organizations offer support to pregnant adolescent girls.

Our data show that the severely depressed participants were more vulnerable to substance abuse. Given the exposure to substances and early sexual activity, we also wondered if the relationship with the male partner was thoughtfully considered or if it the relationship got developed when adolescents were sad, low, needing support or even when they were inebriated. The absence of male partner and family support in addition to the paucity of support networks indicates that the adolescents may not be making sound decisions and relationship choices. The ethical dilemmas and the value judgment in asking questions around substances to the young pregnant adolescent are significant, and we had to be cautious in not being judgmental about their behavior and choices.

### Adolescent mental health and sexual-reproductive health implementation issues

Adolescents in Kenya constitute about 24% of the total population (about 9.2 million) [24, 45], and a focus on integrating adolescent health in national programming has become an important priority for the government. In 2013, the Kenyan government waived user fee for primary health care and boosted the Beyond Zero program-an infant survival program. At a political level, an ambitious devolution initiative has taken place that purports to address the major demand and supply side challenges. With devolution, the hope is that the national and county levels would have clear roles and responsibilities. There are, however, still challenges with expanding services with limited resources, optimizing strategic choices, service integration and collaborative care, especially for maternal, child and adolescent health. The maternal and child health facilities in Kenya have reported slow progress in tackling maternal health. Issues such as unsafe abortion and obstetric complications such as severe bleeding, infection, hypertensive disorders, and obstructed labor pose big challenge. Nearly, 47% of women below ages of 20 had births from unintended pregnancies and nearly 52% of babies delivered for women this age range were by unskilled attendant [3]. An implementation assessment report of Ministry of Health finds that cultural practices, gender norms, religious beliefs, forced migration, poverty and unemployment hinder implementation of adolescent reproductive health practices. Four distinct gaps in the MCH, reproductive health and adolescent health service provision are worth noting: (a) *Inequitable coverage among* certain areas or population groups, especially *adolescents,* (b) *Demand side barriers* that compromise access and utilization of proven high impact interventions. These barriers include long distances to health facilities, high costs, sociocultural and religious beliefs and practices and low status of women as well as lack of knowledge and information. The demand side barriers get further aggravated by provider attitudes, poor quality and limited integration of services that also hamper and discourage utilization of services, (c) *Supply side challenges* emanate from suboptimal functioning of the health systems (infrastructure, human resources for health (HRH), supply chain, health financing, health Information, and leadership/governance); added to this are challenges with staff knowledge, awareness, approach and readiness to embrace change and empirically supported practices, (d) *high burden of HIV and AIDS-related morbidity and mortality* further compound the situation on ground. We would like to add a less explored barrier of (e) *widespread substance abuse and dependency* and access to illicit substances by adolescents in low resource informal settlements. Child and adolescent reproductive health and adolescent mental health coverage in Kenya in primary health service settings need strengthening. Government expenditure on mental health programming and advocacy is negligible. No formal record of expenditure on promotional and preventive mental health, primary health care advocacy exists. The recruitment of psychologists or social workers in mental health facilities are equally neglected issues. While the national legislative framework champions UN SDG principal of universal access to health, free care to vulnerable populations and equitable quality of care, mental health is amongst the most underrepresented domain. Adolescents and young people in Kenya live amidst complex and challenging social environments and limited resources, with over 56% of its population living in poverty [44]. This is also a setting where mental health is highly neglected area with very few practitioners on ground and until today less than 1% of all health care spending in Kenya is devoted to mental health and there are only 0.2 psychiatrists and 0.01 psychologists per 100,000 people [41]. Limited access of adolescent mothers to reproductive health services also predisposes them to higher risks of illness and death. Understanding the reproductive health needs and rights of adolescents is, therefore, central to improving the health services offered to this vulnerable group [24]. The girls most likely to have a live birth before the age of 18 reside in rural and remote areas, or in urban informal settlements, have little or no education, and live in the poorest households [27]. This is an issue that impacts low resource contexts the most, and it is an issue that needs immediate redressal. A 2005 report by the Ministry of Health shows that an estimated half of all pregnancies of women aged 15 to 19 are terminated or end up with serious medical complications. Kenya’s Constitution including the 2007 Kenyan reproductive health policy also protects the rights of adolescents to health care and guarantees protection from abuse and neglect, all forms of violence, harmful cultural practices, inhuman treatment and punishment, and hazardous or exploitative labor—Article 53 and access to free youth friendly health services [46]. Despite these policy stipulations, on ground, there is limited evidence of this as only 12% of health facilities actually provide the recommended comprehensive reproductive health services to adolescents. Mental health coverage is more limited with very few practitioners on ground and the paucity of formally employed social workers, community health extension workers and unskilled and unharnessed potential of the community health volunteers and community health committees makes the adolescent mental health burden even deeper. The significance of adolescent mental health disorders over the lifespan has only recently caught attention and remains a relatively understudied area in global health [41]. Primary health care facilities have taken on the burden of ensuring the child well-being among the local communities. The 2008/2009 KDHS reports that the proportion of births attended by skilled personnel is about 44%, though wide disparities exist across different regions. This proportion is just a 2% point increase from 42% recorded by the KDHS in 2003 [24]. Another gap is the transition from child health services to adult services as adolescents get caught between this with limited to no adolescent friendly services on ground. Early intervention and adolescent friendly services have caught attention in high income countries but remain an area requiring considerably more services and training in LMIC. Evaluation studies done worldwide have noted that very limited interventions have been tested to address adolescent mental health [11]. Those that been used, their effectiveness for high risk or for differential cultural or socioeconomic contexts have not been sufficiently demonstrated. These findings suggest that socio-culturally relevant, psychosocial outreach services are urgently needed. In our formative work, we found that while the health workers, teachers, social workers, and mental health researchers acknowledge the vulnerability of this group, the concerned professionals, educational and health systems in which they function have very limited knowledge, capacity or consensus on outcomes for adolescent health. Identification of critical service, knowledge, community and implementation barriers that stifle the abilities of primary care and community settings in addressing pregnancy in adolescence and their psychosocial and health needs is, therefore, critical. In addition to this, understanding the experiences and barriers before the pregnant adolescents better and ensuring that the desired psychosocial and health outcomes reflected by adolescent stakeholder dovetail with other stakeholder’s care and treatment preferences are important implementation questions. Increasing social protection of this vulnerable group through community and community leadership, healthcare workforce engagement is crucial and requires narrowing community and institutional gaps through participatory action. Of importance here is a need for collective-research, advocacy and policy mobilization on increasing government’s responsibility towards women and adolescent girls’ health. Currently, there is a lack of specific interventions in the region that addresses the needs of pregnant and parenting adolescents. Our review of psychosocial interventions and evidence-based interventions offered in SSA context underscores the need to re-calibrate the system gaps, ‘out of joint’ functioning and communication, knowledge and capacity barriers between stakeholders along with testing efficacy and applicability of these interventions.

## Limitations

This was a cross-sectional study looking at depression and substance use patterns in pregnant adolescents in a peri-urban primary health facility of Nairobi. As a result of its cross-sectional design, we cannot make causal associations between depression and substance abuse. We did not probe further about psychoactive substance once an adolescent denied taking any. We did not want to make our participants to think that we were judging them. We could not do a clinical assessment to further validate the findings of our survey. Future studies may be able to take this factor into account and institute more robust assessment measures for depression, social support and substance use.

## Conclusion

We found a high prevalence of depression, alcohol and other psychoactive abuse in our sample. The noteworthy finding was that the greater the depression the higher the likelihood of comorbid substance abuse in this peripartum adolescents. The existing health and social disparities experienced by pregnant/parenting adolescents in Kenya make it challenging to put one-dimensional interventions in place. The implementation of existing interventions is challenging given that there are additional risks such as exposure to violence, HIV/STIs, food insecurity, school drop outs, poor skills and knowledge and low support from male partners or family. The context of early pregnancy adds to the existing burden of diseases and Disability Adjusted Living Years (DALYs). Addressing perinatal mental health burden in adolescents requires us to develop interventions that are culturally relevant, embedded within the local system and structures, and community-based awareness around responsible SRH for both young men and women is promoted. Implementation issues such as strong leadership on mental health, substance abuse and SRH which would address policy gaps as well as the communication flow between different stakeholders on ground would be critical to address these comorbidities.
